# Retraction Note: A mechanistic model of the neural entropy increase elicited by psychedelic drugs

**DOI:** 10.1038/s41598-022-20093-y

**Published:** 2022-09-15

**Authors:** Rubén Herzog, Pedro A. M. Mediano, Fernando E. Rosas, Robin Carhart‑Harris, Yonatan Sanz Perl, Enzo Tagliazucchi, Rodrigo Cofre

**Affiliations:** 1 Centro Interdisciplinario de Neurociencia de Valparaíso, Universidad de Valparaíso, Pje Harrington 287, 2360103 Valparaiso, Chile; 2 Department of Psychology, University of Cambridge, Cambridge, CB2 3EB UK; 3 Department of Brain Science, Centre for Psychedelic Research, Imperial College London, London, SW7 2DD UK; 4 Data Science Institute, Imperial College London, London, SW7 2AZ UK; 5 Centre for Complexity Science, Imperial College London, London, SW7 2AZ UK; 6 Buenos Aires Physics Institute and Physics Department, University of Buenos Aires, Buenos Aires, Argentina; 7 National Scientific and Technical Research Council, Buenos Aires, Argentina; 8 Universidad de San Andres, Buenos Aires, Argentina; 9 CIMFAV‑Ingemat, Facultad de Ingeniería, Universidad de Valparaíso, Valparaiso, Chile

Retraction of: *Scientific Reports* 10.1038/s41598-020-74060-6, published online 20 October 2020

The Authors have retracted this Article.

After publication, it was brought to the Author's attention that there was a typo in the script used to calculate the differential entropy. Therefore, the reported entropy estimations are invalid and do not produce the expected increase in entropy when using the neural mass model presented in this Article. The Authors recognise this error and apologise for the confusion it may have caused. The Authors are currently working on a corrected version of the model, which includes the activation of the 5HT2A receptor on both excitatory and inhibitory pools; and will test whether it reproduces the entropy increase.

All Authors agree with this retraction and its wording.

